# Dementia Strategies of England and Glasgow Declaration: What Requires to be Done?

**DOI:** 10.1002/alz.084873

**Published:** 2025-01-09

**Authors:** Smruti Bulsari

**Affiliations:** ^1^ University of Essex, Colchester, Essex United Kingdom

## Abstract

**Background:**

Considering the enormity and pace of spread of dementia in Europe, Alzheimer’s Society (Europe) launched the Glasgow Declaration in 2014, giving these fundamental rights to persons living with dementia: timely diagnosis and diagnostic support, person‐centred quality care, equitable access to treatment and being respected as an individual. The objective of this study is to identify the focus areas of the dementia strategies of England, and whether the focus has changed over time. This study also examines how well the focus areas of Prime Minister’s Challenges 2015 and 2020 to ensure the accordance of Glasgow declaration rights.

**Method:**

National Dementia Strategy 2009, Prime Minister’s Challenge on Dementia 2015 and 2020 are downloaded from the repository of dementia strategies of Alzheimer’s Society. Text from these documents is cleaned to remove the headers, footers, page numbers, table of contents and references. The text is then preprocessed, which includes removal of special characters, numbers, words tokenisation, part‐of‐speech tagging and lemmatisation, and removal of stop words, extra spaces and punctuations. This preprocessed text is then used to find the word frequencies, separately for each document. Hierarchical Dirichlet Process (HDP), Bayesian approach, is applied to the text of dementia strategies to identify the key focus areas of each of these strategies.

**Result:**

All the three strategies have one common focus area: care and support for people with dementia. Research is an additional focus area in the 2020 strategy. The results of HDP support the findings based on the word frequencies; for all the three documents, care, support and health of people with dementia has consistently retained the key focus of these strategies throughout.

**Conclusion:**

Dementia strategies have a consistent focus on care and support for people with dementia since the first strategy in 2009. These align with two of the rights of Glasgow declaration, for persons living with dementia. Despite that Glasgow declaration was launched five years after the first dementia strategy of England, the later strategies (2015 and 2020) continue to lack focus on the rights to timely diagnosis, equitable access to treatment and the right to be respected as an individual.

